# The Simultaneous Administration of a Probiotic or Prebiotic with Live *Salmonella* Vaccine Improves Growth Performance and Reduces Fecal Shedding of the Bacterium in *Salmonella*-Challenged Broilers

**DOI:** 10.3390/ani10010070

**Published:** 2019-12-30

**Authors:** Nahed A. El-Shall, Ashraf M. Awad, Mohamed E. Abd El-Hack, Mohammed A. E. Naiel, Sarah I. Othman, Ahmed A. Allam, Mahmoud E. Sedeik

**Affiliations:** 1 Department of Poultry and Fish Diseases, Faculty of Veterinary Medicine, Alexandria University, Alexandria 22758, Egypt; dr.ashrafawad@gmail.com; 2 Poultry Department, Faculty of Agriculture, Zagazig University, Zagazig 44511, Egypt; dr.mohamed.e.abdalhaq@gmail.com; 3 Department of Animal Production, Faculty of Agriculture, Zagazig University, Zagazig 44511, Egypt; Mohammednaiel.1984@gmail.com; 4 Biology Department, College of Science, Princess Nourah Bint Abdulrahman University, BO. Box 24428, Riyadh 11671, Saudi Arabia; sialothman@pnu.edu.sa; 5 Department of Zoology, Faculty of Science, Beni-Suef University, Beni-Suef 65211, Egypt; allam1081981@yahoo.com

**Keywords:** Salmonellosis, Enteritidis, probiotic, prebiotic, broilers

## Abstract

**Simple Summary:**

The current study was performed to study the efficacy of live *Salmonella Enteritidis* (SE) vaccine alone and with simultaneous administration of probiotic or prebiotic on broiler chickens. The using of probiotic or prebiotic products administered via drinking water enhanced the growth performance of broiler chickens experimentally challenged with SE at the 28th day of age. The use of probiotic or prebiotic simultaneously with the live *Salmonella* vaccine can diminish the negative effect of live vaccines in terms of the growth performance, mortality rate, fecal shedding and organ re-isolation of SE. It is therefore a good strategy to relieve the negative effect of the harmful bacterium and improve the growth performance of broilers.

**Abstract:**

Salmonellosis is one of the most important bacterial diseases in poultry, causing heavy economic losses, increased mortality and reduced production. The aim of this study was the comparative efficacy of a commercial probiotic and/or prebiotic with a live attenuated *Salmonella Enteritidis* (SE) vaccine on the protection of broiler chickens from SE challenge. The efficacy of probiotic or prebiotic products, as well as a live *Salmonella Enteritidis* (SE) vaccine at the 7th day of age, administered via drinking water, were evaluated for clinical protection and effects on growth performance of broiler chickens experimentally challenged with SE at the 28th day of age. The use of probiotic or prebiotic simultaneously with the live *Salmonella* vaccine can diminish the negative effect of live vaccine growth performance, reducing mortality rate, fecal shedding, and re-isolation of SE from liver, spleen, heart and cecum. The use of probiotic or prebiotic simultaneously with the application of the live *Salmonella* vaccine is a good practice to diminish the negative effect of the harmful bacteria and improve the growth performance of broilers. Thus, further studies may be carried out with layers and breeders.

## 1. Introduction

*Salmonella* infection is one of the most critical bacterial diseases affecting poultry, resulting in a high mortality rate and production losses. Moreover, it is one of the most common foodborne bacterial diseases for humans worldwide, especially *Salmonella enteritidis* and *Salmonella typhimurium* [1], including more than 2600 serovars belonging to *S. enterica* which are Gram-negative and facultative anaerobes belonging to the family Enterobacteriaceae [2]. Cha et al. [3] reported that *Salmonella* infection is widely distributed in poultry in developing countries. It can be isolated more frequently from chicken litter or fecal samples and its incidence rate can range from 0 to 100% [4]. Hence, its effective prevention and control is a necessary measure.

There are various common control measures to reduce or prevent *Salmonella* organisms’ colonization of the poultry intestinal systems, including feed additives, probiotic or organic acid supplementation of drinking water and the use of vaccines [5]. Inactivated and/or live attenuated vaccines are used to prevent poultry infection with *Salmonella* organisms through promoting acquired immunity [6]. *Salmonella* vaccination has been proven to have some advantages that include reduction of transmission rate, either horizontal or vertical, of *Salmonella* among broiler breeder and/or broiler chicks [7], lowering the prevalence rate of contaminated table eggs with this pathogen [8] and improving survival rate [9]. Nevertheless, Berghaus et al. [10] reported that this measure cannot decrease the environmental contamination of a breeder farm with *Salmonella*.

Competitive exclusion (CE) through the use of probiotics is another technique for preventing *Salmonella* infections in newly hatched chicks [11]. Its mechanism of action depends on the rapid substitution of intestinal microflora by a culture of specific living microorganisms, primarily *Lactobacillus* spp. [12] to produce immediate resistance against field pathogen colonization. Moreover, the administration of the *Lactobacillus* spp. culture can modulate cytokine gene expression induced by *Salmonella*, and thus reduce its pathogenicity in chickens [13]. Conversely, a non-viable component of food, termed prebiotic, provides binding sites of pathogenic bacteria to be flushed out the gut [14]. Glucomannan, is an example for this type of preventive measure, which is an extracted polysaccharide from *Saccharomyces cerevisiae* cell wall. Not only does it prevent adhesion of pathogenic bacteria such as *Salmonella* and *Escherichia coli* to the enterocyte membranes and stimulate an immune response, it also modulates the intestinal microflora inducing a positive effect for bird performance [15,16].

Moreover, the use of probiotics and prebiotics, as well as their combinations, has proven to have beneficial health effects in poultry production [17,18,19]. The aim of this study was the comparative efficacy of a commercial probiotic or prebiotic and their combination with a live attenuated *Salmonella Enteritidis* (SE) vaccine on the protection of broiler chickens experimentally challenged with SE.

## 2. Materials and Methods 

This study was conducted according to the suggestions and guidelines of the advisory group on the ethics of animal experiments at Alexandria University, Egypt.

### 2.1. Salmonella Vaccine: Avipro^®^ Salmonella Vac E

A live lyophilized attenuated vaccine, *Salmonella enteritidis*, strain Sm24/Rif 12/Ssq, (Elanco Co., Cuxhaven, Germany) is a mutant strain derived from metabolic alterations (Metabolic Drift Mutants) (EFSA, 2004) and cited by Bérto [20]. It was administered via drinking water to the chicks, at the 7th day of age, as one dose, containing at least 10^8^ CFU, according to manufacturer instructions.

### 2.2. Probiotic and Prebiotic

A commercial probiotic composed of *Lactobacillus acidophilus*, 1.0 × 10^9^ CFU; *Lactobacillus plantarum*, 1.0 × 10^9^ CFU; *Pediococcus pentosaceus*, 1.0 × 10^9^ CFU; *Saccharomyces cerevisiae*, 1.0 × 10^9^ CFU; *Bacillus subtilis*, 1.0 × 10^9^ CFU; *Bacillus licheniformis*, 1.0 × 10^9^ CFU on 1000 mL deionized water, that was manufactured by K.M.P. Biotech. Co., LTD, Chonburi, Thailand, was added to drinking water according to the manufacturer’s instructions as follows: 1 mL per 5 L from one day until two weeks daily, and at the third week for only three successive days and stopped for four days, while adding 1 mL per 10 L at the fourth week until the end of the experiment.

A commercial prebiotic product containing 2.6 Beta LevaFructan 100 gm on 1000 mL distilled water, manufactured by Gencore INT. INC. Ann Arbor, MI, USA., was added at the rate of 0.5 mL per liter of drinking water from one day until two weeks daily, and at the third week for only three successive days and stopped for four days, while daily adding from the fourth week until the end of the experiment.

### 2.3. The Challenge Bacterium

An isolate of SE from newly hatched chicks was serotyped and identified by ERIC PCR in the study by Sedeik et al. [21]. It was centrifuged at 3000 rpm for 10 min and the sediment was diluted with sterile phosphate buffer saline and adjusted to contain 10^9^ CFU/mL using a McFerland matching tube to be used for challenging. According to the method of Timms [22], the challenge inoculum was prepared at 28 days of age, each bird in the challenged groups was infected orally with 0.5 mL suspension containing 10^9^ CFU/mL SE [23].

### 2.4. Experimental Design

A total of 192 one-day-old broiler (Cobb) chicks were divided into 8 groups (24 chicks each) of three replicates (*n* = 8) as follows: G1: negative control (none treated and none challenged); (G2): positive control (challenged with SE); (G3): probiotic treated and challenged; (G4): prebiotic treated and challenged, (G5): vaccinated and challenged; (G6): vaccinated plus probiotic and challenged; (G7): vaccinated plus prebiotic and challenged; and (G8): vaccinated and not challenged.

The commercial balanced ration that met the broiler chicken requirements according to the National Research Council [24] was used as follows: starter (23% crude protein and metabolizable energy 3008 Kcal/kg), grower (21% crude protein and 3080 Kcal/kg diet) and finisher feed (19% crude protein and 3190 Kcal/kg diet) until 12, 26 and 42 days of age, respectively. All the birds were offered ad libitum feed and water. They were received at 32 °C, which decreased by 2 °C per week until it reached 24 °C, in separated floor pens that had electrical heating and lighting.

### 2.5. Evaluated Parameters

#### 2.5.1. Clinical Investigation and Mortality Rate 

Birds in the challenged groups were observed daily for two weeks post-challenge until the end of the study (6 weeks of age) for clinical signs or death. Dead birds were subjected to necropsy for recording the lesions of SE [25].

#### 2.5.2. The Shedding Rate of SE on Cloacal Swabs

Cloacal swabs were taken from chicks individually in each group at arrival and just before experimental infection (at 28 days of age) and examined bacteriologically to ensure that the birds were free from SE. At days 3, 7 and 14 post-challenge, cloacal swabs were collected from all birds in each group individually on a 10 mL tube of tetrathionate broth and incubated overnight at 37 °C. A loopful from the broth was streaked on *Salmonella shigella* (SS) agar for *Salmonella* isolation. Suspected colonies were identified morphologically and biochemically, as described by Cherry et al. [26].

#### 2.5.3. Re-Isolation Rate of SE from Different Organs

Nine birds from each group (3/replicate) were randomly selected weekly post-challenge, sacrificed, and then the spleen, heart, cecum and liver were collected for SE re-isolation. The homogenized tissue samples were incubated overnight in 1% peptone broth then 1 mL suspension was added to 9 mL tetrathionate broth and incubated at 37 °C for 24 h. A total of 200 µL suspension was sub-cultured on SS agar and incubated for 24 h at 37 °C. Morphological and biochemical identification of suspected colonies was performed.

#### 2.5.4. The Growth Performance

Chickens were weighed individually in each replicate and then the birds in each group were subjected to weekly determination of the production parameters that included body weight gain, feed consumption and feed conversion ratio (FCR).

### 2.6. Statistical Analysis

Statistical analysis of obtained results was carried out according to the Petrie and Watson [27] model and the experimental unit. The values of *p* ≤ 0.05 were considered significant. The statistical analysis was performed using SPSS program (version: 22).

## 3. Results and Discussion

After experimental infection of broiler chickens at 28 days old with SE, the observed clinical signs were depression, ruffled feathers, loss of appetite and watery diarrhea started at the 3rd day post-challenge (PC), with a morbidity rate of 70.8, 16.6 and 20.8% in infected control, probiotic treated and prebiotic treated groups, respectively. However, vaccinated birds with or without probiotic or prebiotic did not exhibit clinical signs or mortality. Most of the mortality occurred during the first week PC, which decreased during the second week PC. Similar results were obtained by Abd El-Ghany et al. [28], although they recorded deaths in the third week PC, and our study was terminated at two weeks PC (42 days of age). 

Positive control birds had the highest mortality rate, which was 25% (6/24 birds), although the same dose of SE resulted in 30.67% deaths on positive control birds by Abd El-Ghany et al. [28], who performed the infection at 20 days of age. The difference of challenging age may be the cause of the different results, as the susceptibility of chickens to infection with *Salmonella* is age-dependent [29] and the chicken breed used may also play a role. 

**Figure 1:**
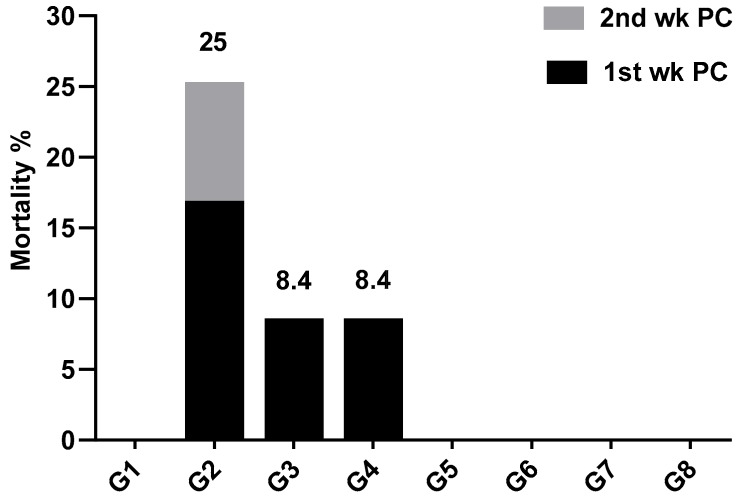
Mortality percentage during two weeks post challenge (PC) with *Salmonella Enteritidis* (SE). wk—week; G—group.

The administration of probiotic or prebiotic starting from 1 day old until SE challenge at 28 days old decreased mortality rate caused by SE challenge to 8.4% (2/24). Moreover, death occurred only during the first week PC, with protection 91.67% on both groups (Figure 1). Abd El-Ghany et al. [28] obtained 12% mortality rate by using probiotic that occurred in the first and second week PC, that could be explained by the fact that they first used the probiotic at 5 days of age and performed the challenge at 21 days of age, while in this study, the probiotic was started from 1 day old and used daily from the challenge day (28 days of age) until the end of the experiment.

The use of live SE vaccine, either with or without probiotic and prebiotic, completely prevented death due to SE challenge (100% protection). Throughout the experiment, negative control and vaccinated control groups did not exhibit any clinical signs or mortality.

**Figure 2:**
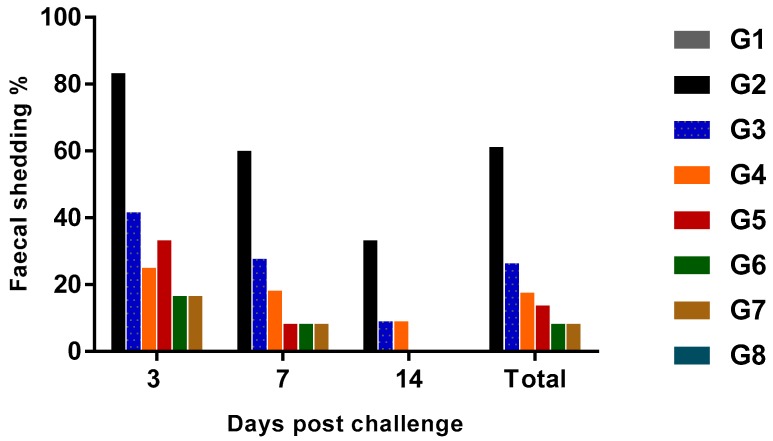
The fecal shedding rate (%) of SE in experimentally challenged groups (*n* = 24).

Horizontal transmission is one of the most prominent routes of *Salmonella* infection in chicken flocks [30] and once bacterial colonization on cecal tonsils occurs, *Salmonella* shedding is consistently observed in the feces [31]. In this study, gradual decrease of the shedding rate of SE on cloacal swabs was observed within each group until the end of the observation period (14th day PC) (Figure 2) even on positive control birds, although it was higher than other groups, as 20/24 birds (83.3%) were positive for SE on the third day PC, which decreased to 60% (12/20) at the 7th day PC and became 33.3% (6/18) at the 14th day PC. These results agreed with those of Stern [32], who reported that *Salmonella* content on the cecum became low or non-detectable at four weeks post-challenge in unvaccinated chickens.

The lowest fecal shedding rate was recorded in vaccinated, and treated with probiotic or prebiotic groups, which were 16.6, 8.4 and 0% at the three times of observation on both groups. The probiotic treated group had 41.6, 27.7 and 9%, the prebiotic treated group had 25, 18.2 and 9% and the vaccinated group had 33.3, 8.4 and 0% at days 3, 7 and 14 PC, respectively. The observable reduced shedding on vaccinated groups with or without prebiotic or probiotic administration, as well as its complete stopping at the 14th day PC may be related mainly to the cell-mediated immunity that was induced by orally administered live *Salmonella* vaccine [33]. Furthermore, the synergistic effect of probiotic or prebiotic with live vaccine was observable, but only at the third day PC.

**Figure 3:**
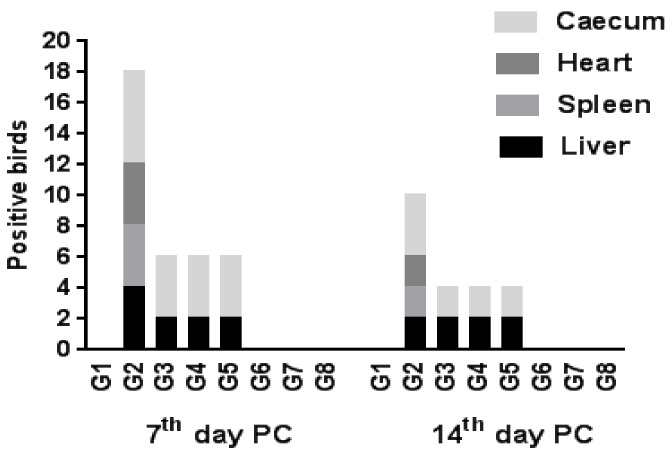
Re-isolation rate of SE from different organs (number of positive birds for each organ/six sacrificed broiler chickens/group).

Regarding SE re-isolation from different internal organs, it was recorded that its highest rate was from the cecum, especially at the 7th day PC on all challenged groups, followed by the liver. Nevertheless, the liver, spleen and heart were equal for SE re-isolation rates on the positive control group, following the cecum. The use of the live vaccine, probiotic or prebiotic each alone decreased the re-isolation rate of the bacterium, particularly from the spleen and heart, to zero, while a combination of the vaccine with probiotic or prebiotic resulted in zero rates on all investigated organs at the 7th day PC (Figure 3, Table 1). These results indicated that the combination of probiotic or prebiotic with live vaccine was most effective on clearance of SE, as previous studies have shown that *Salmonella* may persist on internal organs for long periods, such as that which was observed in a layer hen trial by Sharma et al. [34], who reported that *S. typhimurium* was detected on the spleen and liver for 16 weeks post-infection. Moreover, it was thought that *Salmonella* invades with persistent infection in intestinal cells [35], so the use of prebiotics or probiotics could be used to modulate the gut microflora, and thus limit *Salmonella* colonization of birds [36,37].

Capozzo et al. [38] considered that at least moderate persistence of colonization by the immunogenic agent is required for efficient stimulation of immune responses by live vaccines. McWhorter and Chousalkar [35] suggested that the long persistence of a live *Salmonella* vaccine in the bird is essential for horizontal transmission of the vaccine strain within a flock. Nevertheless, SE has not been detected on examined cloacal swabs of vaccinated control birds (G8) at any time of observation, and the re-isolation of SE has also been zero since the age of 31 days. This means that the vaccine does not persist within the host, as observed by Methner et al. [39] for the SE deletion mutant, which is significantly reduced between seven and 14 days, following infection. So, a further study is required to study the efficacy of this vaccine on long-aged poultry flocks, such as layers and breeders.

Results from the growth performance of different experimental groups including body weight gain and feed conversion ratio (FCR) were reported at intervals of 0–4 weeks (before the challenge), 4–6 weeks (2 weeks after the challenge) and 0–6 weeks (within the experiment) (Table 2).

At 0–4 weeks of age (before the challenge), the use of probiotic and prebiotic on G3 and G4, respectively, had improved body weight gain and FCR (*p* > 0.05) in comparison to non-treated control birds (G1 and G2). This result disagrees with Tellez et al. [40], who reported that growth of probiotic supplemented birds was not significantly different from the non-treated group. In agreement with the present findings, turkeys treated with Mannan-oligosaccharides (MOS) in the feed had significantly more body weight gain than the controls [41]. During the 2 weeks post-challenge and overall period (0–6 weeks) they significantly (*p* < 0.05) improved body weight gain and FCR, in comparison to the positive control birds, but they did not prevent death totally. Similar results were reported by Abd El-Ghany et al. [28], Attia, et al. [42], and Fairchild et al. [43], as they observed that challenging birds with *E. coli* and receiving MOS resulted in significantly greater body weights compared to the untreated birds. Also, Ellakany et al. [44] reported improvement of growth performance with prebiotic on broiler chicks challenged with SE.

The observable positive effect of prebiotic on SE challenge in this study may be attributed to the production of H_2_S_2_ and lactic acid through fermentation of these nutritional components by the beneficial intestinal microflora, and thus decrease the intestinal pH that inhibits the growth of pathogenic bacteria [45]. Probiotic also may act as an inhibitor to pathogenic microorganisms through competitive exclusion and by decreasing the intestinal pH, thus stimulating the growth rate [46]. Nevertheless, Attia et al. [42] found no significant growth improvement with prebiotic on SE challenged chicks. These variable results between different authors may be explained by the difference of probiotic or prebiotic components, dose and the duration of their application.

The significant difference (*p* < 0.5) of body weight gain and FCR results at the period 0–4 weeks of age (before SE challenge) on vaccinated groups either challenged or not, when compared with G1 (none vaccinated) indicated that the live *Salmonella* vaccine had a negative effect on growth performance during the first four weeks of age. However, this negative effect quickly faded by compensatory growth of birds in the vaccinated group (G8), during the fifth and sixth week of age, reflecting a higher overall weight gain, but a still poor FCR. After the challenge for two weeks, the body weight gain of the vaccinated birds (1327 ± 49.7) was significantly higher (*p* < 0.05) than that of the positive control birds (1083 ± 34.1). The FCR of the former was improved (*p* > 0.05) (1.43 ± 0.05) when compared with either the positive control group (1.6 ± 0.05) or negative control group (1.25 ± 0.03) (Table 2), indicating the protective effect of the live SE vaccine against SE challenge, through the induction of cell-mediated immunity [33].

Furthermore, the combination of probiotic or prebiotic with live SE vaccine improved the body weight gain and FCR significantly (*p <* 0.05) during 0–4 weeks of age, as well as the overall body weight gain (0–6 weeks). The use of probiotic or prebiotic could potentially modulate the intestinal microflora and thus contribute to limiting *Salmonella* colonization of poultry [36], and thus could play a role in diminishing the observed negative effect of live *Salmonella* vaccine in this study. Moreover, this improvement of growth performance induced by using probiotic may be attributed to the synthesis of vitamins [47], production of digestive enzymes and lactic acid [48,49] and stimulation of the host’s appetite [50].

Although, Beirão et al. [51] suggested that simultaneous administration of probiotic or prebiotic with live *Salmonella* vaccines may be an obstacle to acquiring immune response by rapid removing of the immunogenic agent from the host, the recorded results of this study proved that either probiotic or prebiotic together with live *Salmonella* vaccine provided a synergistic protective effect against SE challenge in broiler chickens.

## 4. Conclusions

It was concluded that the use of the live SE vaccine has a higher protective effect against SE challenge than probiotic or prebiotic, especially for growth performance and livability rate. But, simultaneous administration of probiotic or prebiotic with live SE vaccine in broiler chickens has a synergistic effect, not only for growth performance and livability rate, but also decreases fecal shedding and re-isolation of SE from different organs, thus reducing environmental contamination with the bacterium.

## Figures and Tables

**Table 1:** Re-isolation rate of SE from different organs: (number of positive birds for each organ/six sacrificed broiler chickens/group).

Measurements	Treatments								
		G1	G2	G3	G4	G5	G6	G7	G8
7th day PC									
Liver	+/total	0/6	4/6	2/6	2/6	2/6	0/6	0/6	0/6
	%	0	66.67	33.33	33.33	33.33	0	0	0
Spleen	+/total	0/6	4/6	0/6	0/6	0/6	0/6	0/6	0/6
	%	0	66.67	0	0	0	0	0	0
Heart	+/total	0/6	4/6	0/6	0/6	0/6	0/6	0/6	0/6
	%	0	66.67	0	0	0	0	0	0
Cecum	+/total	0/6	6/6	4/6	4/6	4/6	0/6	0/6	0/6
	%	0	100	66.67	66.67	66.67	0	0	0
14th day PC									
Liver	+/total	0/6	2/6	2/6	2/6	2/6	0/6	0/6	0/6
	%	0	33.33	33.33	33.33	33.33	0	0	0
Spleen	+/total	0/6	2/6	0/6	0/6	0/6	0/6	0/6	0/6
	%	0	33.33	0	0	0	0	0	0
Heart	+/total	0/6	2/6	0/6	0/6	0/6	0/6	0/6	0/6
	%	0	33.33	0	0	0	0	0	0
Cecum	+/total	0/6	4/6	2/6	2/6	2/6	0/6	0/6	0/6
	%	0	66.67	33.33	33.33	33.33	0	0	0

PC: post-challenge.

**Table 2:** Growth performance (±SD) for experimental groups before and post SE experimental challenge.

Treatments	Measurements					
	Body weight Gain (g)/bird			FCR (g/g)		
	Weeks of Age					
	0–4 (BC) *	4–6 (PC) **	0–6 (Overall)	0–4 (BC) *	4–6 (PC) **	0–6 (Overall)
G1	1385 ± 31.3 ^bc^	1526 ± 38.6 ^a^	2911 ± 57. 3 ^a^	1.38 ± 0.03 ^bc^	1.25 ± 0.03 ^bc^	1.30 ± 0.03 ^cd^
G2	1411 ± 33.6 ^abc^	1083 ± 34.1 ^c^	2494 ± 65.4 ^c^	1.33 ± 0.03 ^bc^	1.60 ± 0.05 ^a^	1.46 ± 0.04 ^ab^
G3	1444 ± 23.4 ^abc^	1408 ± 30.8 ^ab^	2852 ± 52.5 ^a^	1.25 ± 0.02 ^c^	1.21 ± 0.03 ^c^	1.23 ± 0.02 ^d^
G4	1474 ± 21.6 ^ab^	1477 ± 29.0 ^a^	2951 ± 31.9 ^a^	1.28 ± 0.02 ^c^	1.22 ± 0.03 ^c^	1.25 ± 0.01 ^cd^
G5	1268 ± 37.0 ^d^	1327 ± 49.7 ^ab^	2595 ± 54.0 ^bc^	1.53 ± 0.05 ^a^	1.43 ± 0.05 ^abc^	1.48 ± 0.03 ^a^
G6	1360 ± 34.3 ^c^	1445 ± 62.1 ^a^	2805 ± 35.8 ^ab^	1.42 ± 0.04 ^b^	1.25 ± 0.06 ^bc^	1.32 ± 0.02 ^cd^
G7	1507 ± 23.8 ^a^	1227 ± 35.3 ^bc^	2734 ± 46.5 ^ab^	1.34 ± 0.02 ^bc^	1.37 ± 0.04 ^bc^	1.36 ± 0.02 ^bc^
G8	1276 ± 22.3 ^d^	1366 ± 110 ^ab^	2642 ± 126 ^ab^	1.50 ± 0.02 ^a^	1.46 ± 0.13 ^ab^	1.48 ± 0.06 ^a^

Means within a column without a common superscript letter differ significantly (*p* < 0.05). * BC: before challenge. ** PC: post challenge (The challenge with SE was performed at four weeks of age). FCR: feed conversion ratio. ^a, b, c, d^—within a column without a common superscript letter differ significantly (*p* < 0.05).
